# Atopic Dermatitis and Risk of Inflammatory Bowel Disease in Children: A Systematic Review and Meta-Analysis

**DOI:** 10.7759/cureus.112720

**Published:** 2026-07-15

**Authors:** Agostina Loiacono, Amulya Adusumilli, Kaushikkumar S Barot, Abshiro H Mayow, Bhre C Sumitro, Sonalben Chaudhary, Anastasia Postoev, Neelum Ali

**Affiliations:** 1 Internal Medicine, University of Buenos Aires, Buenos Aires, ARG; 2 Internal Medicine, Mahadevappa Rampure Medical College, Kalaburagi, IND; 3 Pediatrics, Shantabaa Medical College & General Hospital Amreli, Amreli, IND; 4 School of Medicine, St. George’s University School of Medicine, St. George’s, GRD; 5 School of Medicine, St. George's University, True Blue, GRD; 6 Internal Medicine, Zydus Sitapur Hospital, Sitapur, IND; 7 Internal Medicine, Caribbean Medical University, Willemstad, CUW; 8 Internal Medicine, University of Health Sciences, Lahore, PAK

**Keywords:** atopic dermatitis, crohn's disease, inflammatory bowel disease, meta-analysis, paediatric

## Abstract

Atopic dermatitis (AD) is a chronic inflammatory skin condition predominantly affecting children, and emerging evidence suggests a potential association with inflammatory bowel disease (IBD). This systematic review and meta-analysis aimed to quantify the risk of developing IBD, Crohn's disease (CD), and ulcerative colitis (UC) in children with AD compared with those without. A comprehensive search of PubMed/MEDLINE, Embase, Cochrane CENTRAL, Web of Science, and Scopus was conducted from inception to June 2025. Cohort and case-control studies involving children aged 0-18 years with a diagnosis of AD and reporting IBD as an outcome were eligible for inclusion. Study quality was assessed using the Newcastle-Ottawa Scale, and pooled odds ratios (ORs) with 95% confidence intervals (CIs) were calculated using a random-effects model. Four studies were included in the meta-analysis. Pooled analysis demonstrated that children with AD had a significantly increased risk of IBD compared with controls (OR: 1.44; 95% CI: 1.26-1.64), with substantial heterogeneity across studies (I² = 70%). Subgroup analyses revealed elevated risks for both CD (OR: 1.46; 95% CI: 1.13-1.90) and UC (OR: 1.27; 95% CI: 1.02-1.59). Leave-one-out sensitivity analysis confirmed the stability and robustness of these findings. In conclusion, children with AD are at a significantly increased risk of developing IBD, highlighting the need for heightened clinical awareness of gastrointestinal comorbidities in this population and warranting further prospective paediatric research.

## Introduction and background

Atopic dermatitis (AD) is a chronic, relapsing inflammatory skin condition characterised by intense pruritus, eczematous lesions, and impaired epidermal barrier function. It predominantly manifests in early childhood, with approximately 80% of cases presenting before the age of six years [1]. Globally, AD affects an estimated 11.1% of children and adolescents, representing a substantial public health burden, with over 72.4 million paediatric cases recorded in 2021 - a 6.2% increase since 2000 [2-3]. Beyond its cutaneous manifestations, AD is now increasingly recognised as a systemic inflammatory disorder driven by immune dysregulation, particularly a dysregulated T-helper 2 (Th2) cytokine response involving interleukin (IL)-4, IL-13, and IL-31, alongside epidermal barrier disruption and gut-skin axis dysfunction [4-5].

Inflammatory bowel disease (IBD), encompassing Crohn's disease (CD) and ulcerative colitis (UC), is a chronic inflammatory condition of the gastrointestinal tract of uncertain aetiology. The incidence of paediatric-onset IBD has risen significantly over recent decades, with global case numbers among children and adolescents increasing by over 22% between 1990 and 2019 [6-7]. Paediatric IBD is particularly concerning given its earlier onset, more extensive disease at diagnosis, and greater impact on growth and development compared with adult-onset disease [8]. An association between IBD and systemic inflammatory skin disorders has also been described beyond AD, including hidradenitis suppurativa (HS) and psoriasis, both of which share overlapping immune-mediated pathways with IBD [9-10].

The potential association between AD and IBD has garnered considerable interest, given the shared pathophysiological mechanisms between the two conditions, including immune dysregulation, gut microbiota dysbiosis, defective epithelial barrier function, and overlapping genetic susceptibility loci [4,11]. The gut-skin axis - a bidirectional communication network linking gut microbial composition to cutaneous immune responses - has been proposed as a key mechanistic link, whereby systemic Th2 activation in AD may contribute to intestinal barrier disruption and promote chronic gut inflammation [5].

Several studies found that AD was associated with IBD, but most of the studies included adult patients [12-13]. Despite this growing body of evidence, results across paediatric studies remain inconsistent, particularly regarding the risk of UC, and no meta-analysis to date has focused exclusively on children. Therefore, we conducted a systematic review and meta-analysis to quantify the risk of IBD, CD, and UC in children with AD compared with those without.

## Review

Methodology

This study was carried out in accordance with the Preferred Reporting Items for Systematic Reviews and Meta-Analyses (PRISMA) 2020 Statement and its recommended reporting standards [14]. The protocol of this study was registered with PROSPERO (CRD42036625235).

Literature Search and Search Strategy

A comprehensive and systematic search of the literature was conducted across five major electronic databases: PubMed/MEDLINE, Embase, Cochrane Central Register of Controlled Trials (CENTRAL), Web of Science, and Scopus. Searches were performed from database inception through to 20 May 2026, with no restriction on language or publication date. Reference lists of all included studies and relevant review articles were manually screened to identify any additional eligible studies not captured by the electronic search.

The literature search incorporated both Medical Subject Headings (MeSH) and relevant free-text terminology to maximize retrieval of eligible studies. Search terms were combined using appropriate Boolean operators (AND and OR) and were tailored to the indexing structure and search requirements of each database.

("atopic dermatitis" OR "atopic eczema" OR "eczema" OR "atopy") AND ("inflammatory bowel disease" OR "Crohn's disease" OR "Crohn disease" OR "ulcerative colitis" OR "IBD" OR "CD" OR "UC") AND ("child*" OR "paediatric*" OR "pediatric*" OR "infant*" OR "adolescent*" OR "juvenile*" OR "youth" OR "school-age" OR "preschool"). The search was restricted to studies involving human subjects. Detailed search strategies are mentioned in the appendix.

Study Selection and Eligibility Criteria

Studies were screened independently by two reviewers in two sequential phases. In the first phase, titles and abstracts were assessed against the eligibility criteria. In the second phase, full texts of potentially eligible studies were retrieved and evaluated. Discrepancies between reviewers at either phase were resolved through discussion or, where consensus could not be reached, by consultation with a third reviewer.

Regarding inclusion criteria, studies were eligible if they involved children and/or adolescents aged 0-18 years with a diagnosis of atopic dermatitis (AD), provided that studies reporting a paediatric subgroup separately were also considered eligible. The diagnosis of AD was required to be confirmed by clinical assessment, validated diagnostic codes (ICD-9/ICD-10), physician diagnosis, or validated questionnaire criteria such as the UK Working Party criteria. An appropriate comparator group of children without AD was required to serve as the reference population. The primary outcomes of interest were the incidence or risk of developing IBD (overall), CD, or UC as a new diagnosis following the diagnosis of AD. With respect to study design, cohort studies (both prospective and retrospective) and case-control studies were eligible, provided they reported adjusted or unadjusted measures of association.

Case reports, case series, editorials, conference abstracts, and narrative or systematic review articles were excluded, as were studies that reported only the prevalence of IBD among AD patients without an appropriate non-AD comparator group. Studies in which IBD served as the exposure and AD as the outcome were excluded on the basis of reverse temporality. Finally, where duplicate publications reported data from the same study cohort, only the most complete or most recently published report was retained for analysis.

Given the limited number of studies reporting outcomes exclusively in paediatric populations, studies with a mixed adult and paediatric cohort were also considered eligible provided that children constituted at least 50% of the total study population. To assess the potential impact of this decision on the pooled estimates, a leave-one-out sensitivity analysis was conducted, with separate removal of any mixed-population study to confirm that its inclusion did not introduce meaningful age-related bias into the overall findings.

Quality Assessment

The risk of bias and methodological robustness of the included observational studies were independently appraised by two investigators using the Newcastle-Ottawa Scale (NOS), a validated tool designed for assessing cohort and case-control studies [15]. The scale examines study quality within three key areas: participant selection (maximum score of four stars), group comparability based on methodological design or statistical adjustment (maximum of two stars), and ascertainment of either outcomes or exposures (maximum of three stars). Studies receiving a total score between 7 and 9 were classified as having high methodological quality, scores ranging from 4 to 6 indicated moderate quality, and scores of 3 or below were considered indicative of low quality. Any discrepancies in scoring between the reviewers were discussed and reconciled through mutual agreement.

Data Extraction

Data from each eligible study were extracted independently by two reviewers using a standardised, piloted extraction form. Extracted variables included: first author and year of publication; country/region; study design; sample size and number of children with AD; method of IBD/CD/UC ascertainment; and outcome data. Any discrepancies between the two reviewers during data extraction were resolved through discussion, and where agreement could not be reached, a third reviewer was consulted for final adjudication.

Data Analysis

The Review Manager version 5.4.1 was used for conducting the meta‑analysis. To account for the clinical and methodological heterogeneity expected across studies with diverse populations and study designs, a random-effects model using the DerSimonian-Laird method was applied as the primary analytical approach. Effect estimates were pooled as odds ratios (OR) with 95% confidence interval (CI). Separate meta-analyses were conducted for: (i) overall IBD; (ii) CD; and (iii) UC. Statistical heterogeneity across studies was assessed using Cochran's Q test and quantified using the I2 statistic. I2 values of <25%, 25-50%, and >50% were interpreted as low, moderate, and high heterogeneity, respectively. We were unable to perform publication bias as the number of included studies was less than 10.

Results

**Figure 1:**
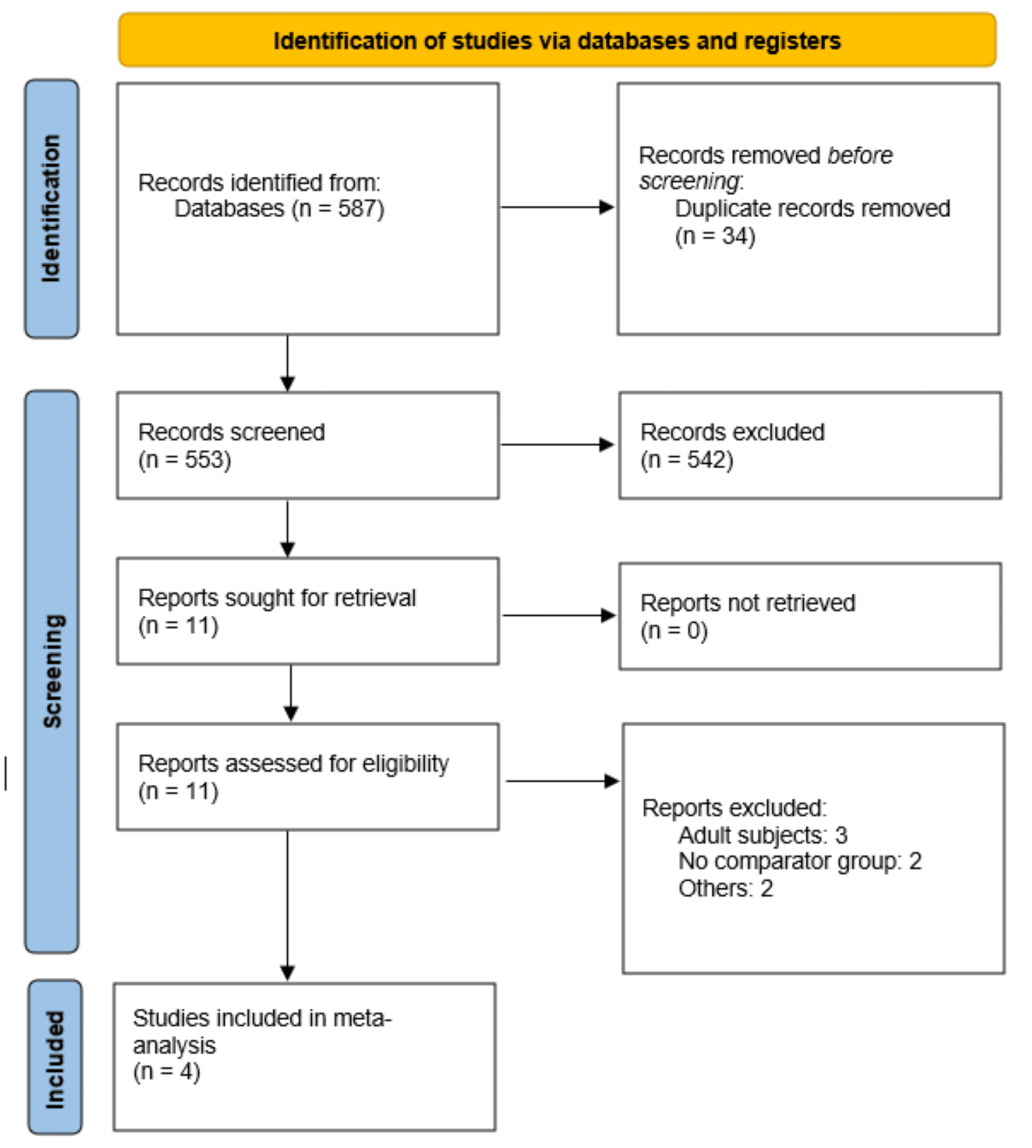
Study selection process (PRISMA Flowchart)

The PRISMA flowchart of study selection is shown in Figure 1. Our systematic literature search identified 553 records after removing duplicates, with 542 excluded after screening the titles and abstracts. Finally, four studies were included in the meta-analysis. Table 1 presents characteristics of included studies. Follow-up duration of included studies ranged from 4 years to 12 years. Table 2 presents quality assessment of included studies.

**Table 1:** Characteristics of included studies AD – Atopic Dermatitis, IBD – Inflammatory Bowel Disease, ICD – International Classification of Diseases, n – Number of Subjects/Participants

Author	Year	Study Design	Region	Subjects with AD (n)	Comparator Subjects	IBD Assessment	Follow-up
Ahn et al. [16]	2024	Cohort	South Korea	39832	159328	ICD Codes	12 Years
Fuxench et al. [17]	2023	Cohort	United Kingdom	409426	1809029	ICD Codes	5 Years
Lerchova et al. [18]	2024	Cohort	Sweden	31671	51640	ICD Codes	NR
Lusignan et al. [19]	2022	Cohort	United Kingdom	173309	694,836	ICD Codes	4.1 Years

**Table 2:** Quality assessment of included studies using New-castle Ottawa Scale (NOS)

Author	Selection	Comparison	Assessment	Overall
Ahn et al. [16]	3	2	2	7
Fuxench et al. [17]	3	2	3	8
Lerchova et al. [18]	3	2	2	7
Lusignan et al. [19]	3	2	3	8

Risk of IBD in Patients with AD

**Figure 2:**
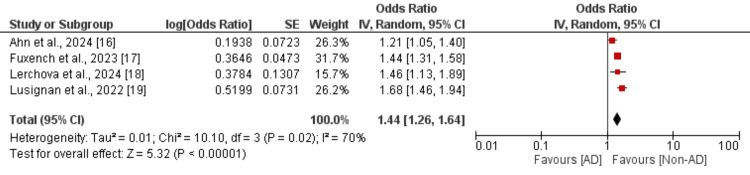
Comparison of IBD between two groups IBD – Inflammatory Bowel Disease

As shown in Figure 2, the meta‑analysis of four studies identified significant associations of AD with IBD (pooled OR: 1.44, 95% CI: 1.26-1.64), and substantial statistical heterogeneity across the included studies was demonstrated (I2 = 70%). Sensitivity analysis was performed by removing one study at a time, and the results are presented in Table 3. The pooled odds ratios remained statistically significant across all iterations, ranging from 1.36 (95% CI: 1.20-1.54; following removal of the Lusignan et al. study) to 1.44 (95% CI: 1.15-1.80; following removal of the Fuxench et al. study), indicating that no single study exerted undue influence on the overall result. Overall, these findings confirm that the pooled association between atopic dermatitis and IBD risk in children was robust and not driven by any individual study. Although Lusignan et al. (2022) included both paediatric and adult patients, children constituted approximately 50% of the total study population; furthermore, the removal of this study in the leave-one-out sensitivity analysis did not materially alter the pooled estimate (OR 1.44; 95% CI: 1.15-1.80), suggesting that its inclusion did not introduce meaningful age-related bias into the overall findings.

**Table 3:** Sensitivity analysis

Study	OR (95% CI)	I-Square
Ahn et al., 2024 [16]	1.42 (1.37 to 1.69)	38%
Fuxench et al., 2023 [17]	1.44 (1.15 to 1.80)	80%
Lerchova et al., 2024 [18]	1.43 (1.22 to 1.68)	80%
Lusignan et al., 2022 [19]	1.36 (1.20 to 1.54)	52%

Risk of UC and CD in Patients with AD

Subgroup analysis by IBD subtype revealed that children with AD had a significantly elevated risk of Crohn's disease compared with those without AD (OR: 1.46; 95% CI: 1.13-1.90). A similarly increased, though comparatively more modest, risk was observed for ulcerative colitis (OR: 1.27; 95% CI: 1.02-1.59), with the confidence interval approaching unity, suggesting a weaker but nonetheless statistically significant association (Table 4). Considerable between-study heterogeneity was observed for both CD and UC outcomes, warranting cautious interpretation of the pooled estimates.

**Table 4:** Subgroup analysis based on type of IBD OR: Odds ratio; CI: Confidence interval; IBD: Inflammatory bowel disease

Study	OR (95% CI)	I-Square
Ulcerative colitis	1.27 (1.02 to 1.59)	63%
Crohn's disease	1.46 (1.13 to 1.90)	85%

Discussion

This meta-analysis assessed the risk of developing IBD in children with AD. We identified evidence showing that AD patients are more likely to have prevalent IBD. The denotation of meta‑analysis indicates that AD patients are associated with increased risk of IBD, including UC and CD. A prior meta-analysis by Yu et al. (2024), which included both adult and paediatric populations across eight retrospective cohort studies, similarly reported that AD was associated with significantly elevated risks of IBD, CD, and UC with high heterogeneity for IBD subtypes [11].

To our knowledge, this is among the first meta-analyses to focus exclusively on a paediatric population, thereby providing a more precise and clinically relevant estimate of this association in children. AD's early onset coincides with maturation of gut immune tolerance, so earlier onset or longer duration may increase cumulative Th2 exposure, though included studies lacked such stratification. This parallels the "atopic march," where cutaneous barrier defects drive progressive multi-organ immune dysregulation, offering a plausible framework for AD-to-IBD progression [12].

The biological plausibility of the AD-IBD association is supported by several shared pathophysiological mechanisms. Both conditions are characterised by dysregulated immune responses, impaired epithelial barrier function, and gut microbiota dysbiosis [20-21]. In AD, epidermal barrier disruption - frequently attributed to loss-of-function mutations in the filaggrin (FLG) gene - facilitates allergen penetration and triggers the release of epithelial-derived alarmins, including thymic stromal lymphopoietin (TSLP), IL-33, and IL-25, which potently activate type 2 innate lymphoid cells and drive Th2 polarisation [22-23]. The resulting systemic Th2 cytokine milieu, characterised by elevated IL-4, IL-13, and IL-5, is not confined to the skin. Via the gut-skin axis - a bidirectional immunological communication network - circulating Th2 cytokines may reach the intestinal mucosa, where they disrupt intestinal barrier integrity, alter microbial composition, and promote chronic mucosal inflammation [8, 11]. The observation that AD severity appears to confer a dose-response relationship with IBD risk further supports a mechanistic causal pathway rather than mere shared genetic susceptibility [17].

The finding that the risk of CD was greater than that of UC in our pooled paediatric analysis is noteworthy and somewhat counterintuitive, given that both AD and UC are classically considered Th2-predominant diseases. This was also observed by Chiesa Fuxench et al. (2023), whose senior author commented that this reversal of the biologically predicted direction was an unexpected finding [17]. This paradox may be explained by the unique immunological state of early childhood. The neonatal and infant gut microbiome is still undergoing establishment, and children with AD frequently exhibit reduced microbial diversity and delayed colonisation by key regulatory taxa such as Bifidobacterium and Faecalibacterium prausnitzii [20]. This early-life dysbiosis coincides with a critical window of immune tolerance development, during which the gut mucosal immune system relies on microbial signals to establish regulatory T-cell populations and maintain barrier homeostasis. Disruption of this process may impair oral tolerance and permit deeper bacterial translocation across the intestinal epithelium, favouring the granulomatous, transmural inflammatory pattern characteristic of CD rather than the superficial, colon-limited inflammation of UC. Additionally, impaired innate immune signalling, including altered pattern-recognition receptor function and epithelial antimicrobial peptide production, both of which have been implicated in AD, may further compromise mucosal defence in the terminal ileum and proximal colon, regions preferentially affected by paediatric CD [21]. While this remains hypothetical, it offers a plausible mechanistic explanation consistent with the gut-skin axis framework, and highlights the need for future studies incorporating microbiome profiling in children with AD to clarify this relationship.

The substantial between-study heterogeneity observed in this meta-analysis (I² = 70% for overall IBD) warrants careful consideration. Heterogeneity likely reflects differences in study design, geographic populations, methods of AD and IBD ascertainment, the use of administrative diagnostic codes versus clinical diagnoses, age ranges of the included paediatric populations, and the extent to which studies adjusted for relevant confounders such as family history of IBD, antibiotic use, and socioeconomic status [15, 24]. The use of treatment exposure as a proxy for AD severity - as employed in the THIN database studies - introduces potential misclassification bias, as prescribing practices vary considerably between physicians and healthcare systems.

It is important to contextualise these relative risk estimates against the low baseline incidence of paediatric IBD. IBD remains an uncommon diagnosis in childhood, with population-level incidence typically reported in the range of a few to around ten cases per 100,000 children per year, depending on region [6-7]. Even a 44% relative increase in risk among children with AD therefore translates into a modest absolute increase in the number of expected IBD cases, and the overwhelming majority of children with AD - including those with moderate-to-severe disease - will never develop IBD. Clinicians should interpret these findings as grounds for maintaining awareness of gastrointestinal symptoms in children with AD, rather than as justification for routine gastroenterological referral or invasive investigation (e.g., endoscopy) in asymptomatic children. Overinterpretation of relative risk data risks generating unnecessary parental anxiety and inappropriate use of specialist and endoscopic resources. Referral should continue to be guided by the presence of specific red-flag gastrointestinal symptoms (e.g., persistent diarrhoea, rectal bleeding, weight loss, growth faltering) rather than AD status alone.

Several limitations of this meta-analysis merit acknowledgement. First, the number of eligible studies focusing exclusively on paediatric populations remains small, limiting the statistical power of subgroup analyses. Second, the majority of included studies relied on administrative health record databases, which may be subject to coding inaccuracies and surveillance bias - children with AD may be more frequently followed up by healthcare professionals, increasing the likelihood of IBD detection compared with the general paediatric population. Third, residual confounding by unmeasured variables, including family history of IBD, diet, antibiotic exposure in infancy, and mode of delivery, cannot be excluded in observational studies. One protocol deviation should be noted: Lusignan et al. (2022) included a mixed sample (approximately 50% children), and pediatric-only data were unavailable. This study was retained due to limited pediatric evidence overall. A sensitivity analysis excluding it showed results remained essentially unchanged. We acknowledge this deviation from our pediatric-only criterion and recommend readers interpret related findings with appropriate caution. Fifth, the relatively modest absolute risk of IBD in children - even in those with AD - should be contextualised when translating these relative risk estimates into clinical practice, as IBD remains an uncommon condition in childhood. Lastly, we were not able to perform subgroup analysis due to the limited number of studies and lack of individual-level data. Given these methodological constraints, the present findings should not be interpreted as a basis for altering current paediatric clinical guidelines, introducing routine screening, or pursuing invasive investigations such as endoscopy in asymptomatic children with AD; rather, they highlight an area warranting further prospective, mechanistically-informed research.

Despite these limitations, the findings of this meta-analysis have important clinical and research implications. Paediatricians and dermatologists managing children with AD, particularly those with moderate-to-severe disease, should maintain a heightened awareness of gastrointestinal symptoms that may herald the development of IBD.

Given that both AD and IBD share overlapping immunological pathways, targeted immunotherapies used in one condition may theoretically offer relevant mechanistic insight for dually affected children; however, translating this overlap into practice requires considerable caution. Dupilumab is approved for paediatric AD from six months of age and for IBD-related indications only in more limited contexts, whereas JAK inhibitors such as upadacitinib carry stricter age and weight thresholds, along with black-box warnings for serious infection, malignancy, major adverse cardiovascular events, and thrombosis - risks that require careful weighing against benefit in children, in whom long-term safety data remain limited. Pharmacokinetic and pharmacodynamic profiles also differ substantially between children and adults, and dosing cannot be directly extrapolated from adult regimens. Any decision to use these agents in a child with both AD and IBD should therefore be individualised, made jointly by paediatric dermatology and gastroenterology specialists, and should not be inferred from shared mechanistic pathways alone. We raise this only to highlight a potential area for future clinical research rather than to suggest a current treatment pathway.

Additionally, future prospective studies with standardised diagnostic criteria, adequate follow-up durations, and representative paediatric cohorts from diverse geographical regions are needed to further characterise the magnitude of this association and to elucidate the specific immunopathological mechanisms driving the gut-skin inflammatory axis in childhood.

## Conclusions

This meta-analysis suggests that atopic dermatitis in children may be associated with an increased risk of developing inflammatory bowel disease, including both Crohn's disease and ulcerative colitis, compared with unaffected peers. Given the observational design of the included studies, substantial between-study heterogeneity (I²=70%), and reliance on administrative diagnostic coding, these findings should be interpreted as hypothesis-generating rather than confirmatory of a causal relationship. Current evidence is insufficient to support changes to existing paediatric clinical guidelines or the introduction of routine gastroenterological screening or endoscopic evaluation in children with AD. Rather, these findings support awareness of gastrointestinal symptoms as a potential, uncommon comorbidity, with referral guided by clinical presentation rather than AD diagnosis alone. Future large-scale prospective studies across diverse paediatric populations are warranted to clarify this association and its clinical relevance.

## Appendices

**Table 5:** Search strategies

Database	Search Strings
PubMed	#1 "Dermatitis, Atopic"[Mesh] OR atopic dermatitis[tiab] OR atopic eczema[tiab] OR eczema[tiab] OR atopy[tiab] #2 "Inflammatory Bowel Diseases"[Mesh] OR "Crohn Disease"[Mesh] OR "Colitis, Ulcerative"[Mesh] OR inflammatory bowel disease[tiab] OR IBD[tiab] OR Crohn*[tiab] OR ulcerative colitis[tiab] #3 "Child"[Mesh] OR "Adolescent"[Mesh] OR "Infant"[Mesh] OR child*[tiab] OR pediatric*[tiab] OR paediatric*[tiab] OR infant*[tiab] OR adolescent*[tiab] OR juvenile*[tiab] OR youth[tiab] #4 #1 AND #2 AND #3 Filters: Humans; no date/language limit; searched inception–20 May 2026
EMBASE	#1 'atopic dermatitis'/exp OR 'atopic dermatitis':ti,ab OR 'atopic eczema':ti,ab OR eczema:ti,ab OR atopy:ti,ab #2 'inflammatory bowel disease'/exp OR 'crohn disease'/exp OR 'ulcerative colitis'/exp OR 'inflammatory bowel disease':ti,ab OR ibd:ti,ab OR crohn*:ti,ab OR 'ulcerative colitis':ti,ab #3 'child'/exp OR 'adolescent'/exp OR 'infant'/exp OR child*:ti,ab OR p?ediatric*:ti,ab OR infant*:ti,ab OR adolescent*:ti,ab OR juvenile*:ti,ab OR youth:ti,ab #4 #1 AND #2 AND #3 Limits: 'human'/de; exclude 'conference abstract'/it; date/language unrestricted; inception–20 May 2026
Cochrane	#1 MeSH descriptor: [Dermatitis, Atopic] explode all trees #2 (atopic dermatitis OR atopic eczema OR eczema OR atopy):ti,ab,kw #3 #1 OR #2 #4 MeSH descriptor: [Inflammatory Bowel Diseases] explode all trees #5 MeSH descriptor: [Crohn Disease] explode all trees #6 MeSH descriptor: [Colitis, Ulcerative] explode all trees #7 (inflammatory bowel disease OR IBD OR Crohn* OR ulcerative colitis):ti,ab,kw #8 #4 OR #5 OR #6 OR #7 #9 MeSH descriptor: [Child] explode all trees #10 MeSH descriptor: [Adolescent] explode all trees #11 MeSH descriptor: [Infant] explode all trees #12 (child* OR p?ediatric* OR infant* OR adolescent* OR juvenile* OR youth):ti,ab,kw #13 #9 OR #10 OR #11 OR #12 #14 #3 AND #8 AND #13
Web of Science	#1 TS=("atopic dermatitis" OR "atopic eczema" OR eczema OR atopy) #2 TS=("inflammatory bowel disease" OR IBD OR "Crohn's disease" OR "Crohn disease" OR "ulcerative colitis") #3 TS=(child* OR p?ediatric* OR infant* OR adolescent* OR juvenile* OR youth) #4 #1 AND #2 AND #3 Timespan: All years–20 May 2026; no language restriction
SCOPUS	TITLE-ABS-KEY("atopic dermatitis" OR "atopic eczema" OR eczema OR atopy) AND TITLE-ABS-KEY("inflammatory bowel disease" OR IBD OR "Crohn's disease" OR "Crohn disease" OR "ulcerative colitis") AND TITLE-ABS-KEY(child* OR p?ediatric* OR infant* OR adolescent* OR juvenile* OR youth) Date range: inception–20 May 2026; no language restriction
